# Robotic and Virtual Reality BCIs Using Spatial Tactile and Auditory Oddball Paradigms

**DOI:** 10.3389/fnbot.2016.00020

**Published:** 2016-12-06

**Authors:** Tomasz M. Rutkowski

**Affiliations:** BCI-LabTokyo, Japan

**Keywords:** brain–computer interface (BCI), robotics, virtual reality, symbiotic brain–robot interaction, auditory BCI, tactile BCI, spatial BCI, information geometry

## Abstract

The paper reviews nine robotic and virtual reality (VR) brain–computer interface (BCI) projects developed by the author, in collaboration with his graduate students, within the BCI–lab research group during its association with University of Tsukuba, Japan. The nine novel approaches are discussed in applications to direct brain-robot and brain-virtual-reality-agent control interfaces using tactile and auditory BCI technologies. The BCI user intentions are decoded from the brainwaves in realtime using a non-invasive electroencephalography (EEG) and they are translated to a symbiotic robot or virtual reality agent thought-based only control. A communication protocol between the BCI output and the robot or the virtual environment is realized in a symbiotic communication scenario using an user datagram protocol (UDP), which constitutes an internet of things (IoT) control scenario. Results obtained from healthy users reproducing simple brain-robot and brain-virtual-agent control tasks in online experiments support the research goal of a possibility to interact with robotic devices and virtual reality agents using symbiotic thought-based BCI technologies. An offline BCI classification accuracy boosting method, using a previously proposed information geometry derived approach, is also discussed in order to further support the reviewed robotic and virtual reality thought-based control paradigms.

## 1. Introduction

A brain-computer interface (BCI) is a neurotechnology application that decodes a central nervous system signals of a user. The BCI allows thus for a direct thought–based communication with other users or a control of various appliances (e.g., a direct brain–robot interface) without any involvement of efferent peripheral nervous system fibers or muscles (Wolpaw and Wolpaw, 2012). The state-of-the-art BCI applications rely mostly on visual (Chang et al., 2013; Aminaka et al., 2015, 2016) stimulus modality. However, tactile (Mori et al., 2013a; Kodama et al., 2014, 2016; Shimizu et al., 2014, 2015c; Rutkowski and Mori, 2015; Yajima et al., 2015) and auditory (Schreuder et al., 2010; Chang et al., 2013, 2014; Nakaizumi et al., 2015a) BCIs offer viable alternatives and in some cases they are the more suitable communication augmentation options in case of locked-in-syndrome (LIS) users who cannot focus or control their eye movements (Patterson and Grabois, 1986; Rutkowski and Mori, 2015). The direct brain–robot and virtual reality (VR) BCIs discussed in this paper are utilizing brain event related responses (ERPs) to tactile or auditory stimuli. The online control of robotic and VR applications still suffers from brainwave decoding flaws. In order to improve the previously developed by the author and his students, robotic as well as VR BCIs (Mori et al., 2013a; Hamada et al., 2014; Kodama et al., 2014, 2016; Neto et al., 2014; Nakaizumi et al., 2015a; Rutkowski and Mori, 2015; Rutkowski and Shinoda, 2015; Rutkowski et al., 2015b; Shimizu et al., 2015c; Yajima et al., 2015) a new information geometry method, proposed by Barachant et al. (2012), is applied in offline processing mode to the EEG signals from the previous online (realtime) experimental projects. The information geometry-based technique has been already successfully applied to a brain sleep apnea automatic classification (Rutkowski, 2016). This novel technique employs the information geometry framework first envisioned by Amari (1997, 2016) and further expanded within Riemannian geometry approaches by Barachant et al. (2012).

From now on the paper is organized as follows. In the next section, the novel information geometry-based classification approach is explained and compared with the previously applied machine learning methods for the VR and robotic BCIs. Discussion and conclusions summarize the paper together with future research remarks.

## 2. Methods

All the nine robotic and VR BCI studies summarized below were carried out in accordance with recommendations and permissions of the Ethical Committee from Faculty of Engineering, Information and Systems at the University of Tsukuba, Japan, and Ethical Committee of RIKEN Brain Science Institute, Japan. All the realtime BCI and EEG acquisition experiments were conducted after the participants signed written informed consents in accordance with the Declaration of Helsinki. The EEG signals from the previously conducted VR and robotic BCI studies (Mori et al., 2013a; Hamada et al., 2014; Kodama et al., 2014, 2016; Neto et al., 2014; Nakaizumi et al., 2015a; Rutkowski and Mori, 2015; Rutkowski and Shinoda, 2015; Rutkowski et al., 2015b; Shimizu et al., 2015c; Yajima et al., 2015) have been uniformly bandpass filtered with cut-off frequencies at 0.1 and 40 Hz, respectively. For the BCI classification accuracy improvement simple comparison. All the signals were additionally uniformly segmented (“epoched”) within 0–1000 ms latencies from the auditory or tactile stimulus onsets.

### 2.1. Brain ERP responses classification for BCI with SWLDA and MDM methods

**Figure 1:**
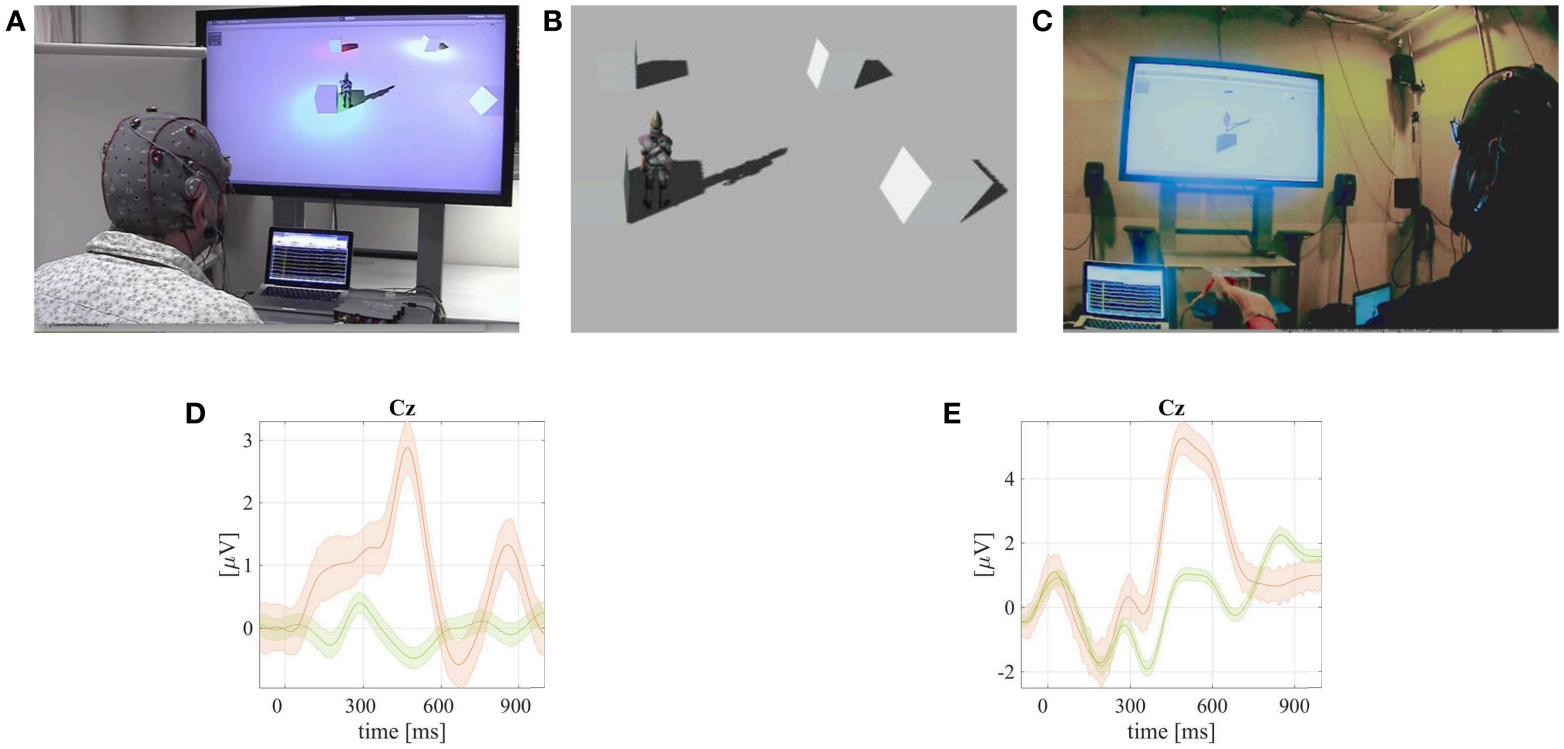
**A screenshot from a video available online^**1**^ that demonstrates the tactile–bone–conduction auditory (VRtbcaBCI) and tactile–push–pressure (VRtppBCI) BCIs for the VR agent control**. Published with permission of the depicted user. The grand mean averaged ERPs with P300 responses (rare targets – orange line; and non–targets green) together with standard error intervals are shown in **(D,E)**. **(A)** The VRtbcaBCI. **(B)** A simple VR scene. **(C)** The VRtppBCI. **(D)** Grand mean averaged ERP of the rare targets with P300 (orange line) and non–targets (green) together with standard error intervals from VRtbcaBCI experiments. **(E)** Grand mean averaged ERP of the rare targets with P300 (orange line) and non–targets (green) together with standard error intervals from VRtppBCI experiments.

**Figure 2:**
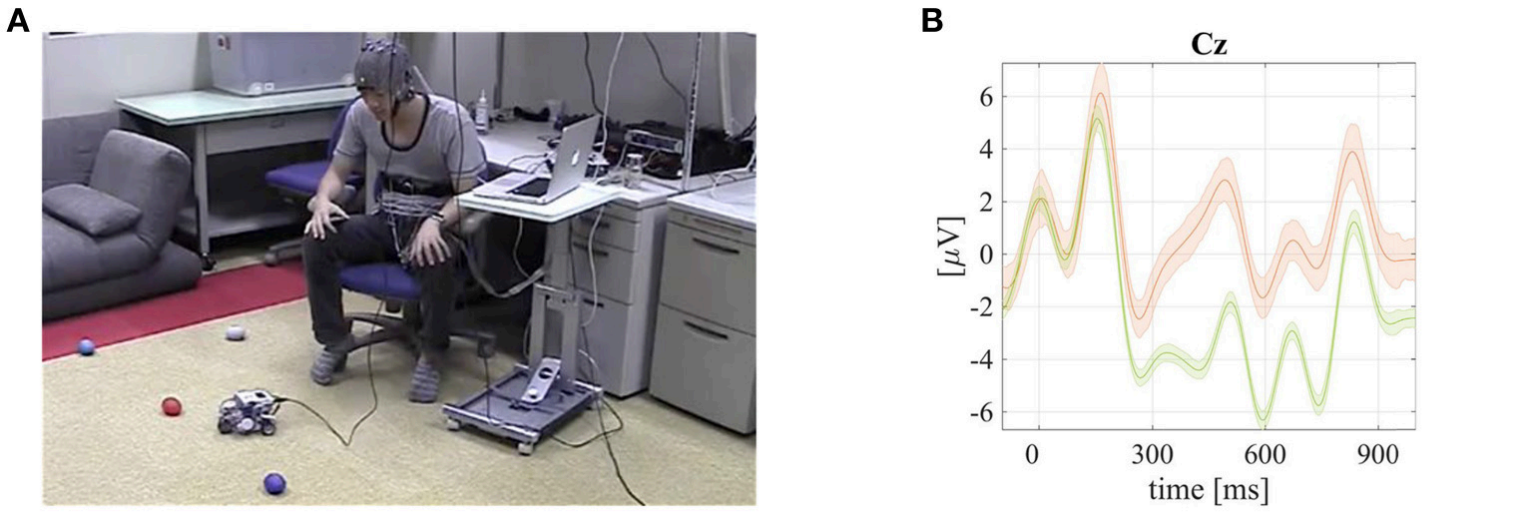
**The RtcBCI experimental set–up (a screenshot from an online demo video^**2**^) and grand mean averaged tactile ERPs with P300 responses visualized**. Published with permission of the depicted user. **(A)** A screenshot from a video available online^2^ that demonstrates the RtcBCI–based small LEGO vehicle robot control. **(B)** Grand mean averaged ERP of the rare targets with P300 (orange line) and non–targets (green) together with standard error intervals from RtcBCI experiments.

**Figure 3:**
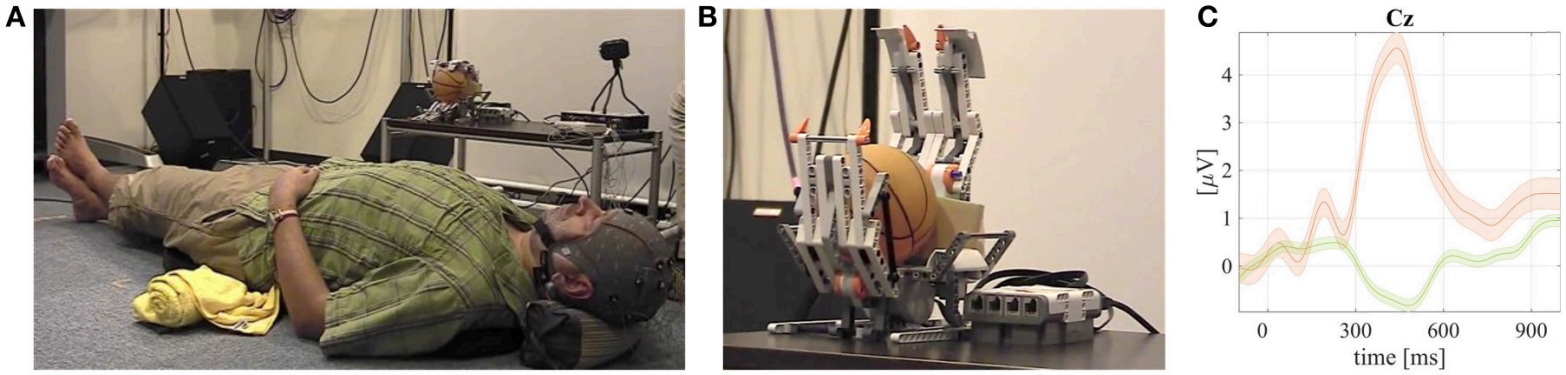
**Screenshots from a video available online^**3**^ that demonstrates the robotic body–tactile (RbtBCI) for the LEGO robotic hand control and grand mean averaged tactile ERPs with P300 responses visualized**. Published with permission of the depicted user. **(A)** A user laying on the vibrotactile pad with embedded tactile stimulators and with the EEG cap on his head. The LEGO robotic prothetic hand model is on a table next to him. **(B)** The LEGO robotic prosthetic hand during the RbtBCI control. **(C)** Grand mean averaged ERP of the rare targets with P300 (orange line) and non–targets (green) together with standard error intervals from RbtBCI experiments.

**Figure 4:**
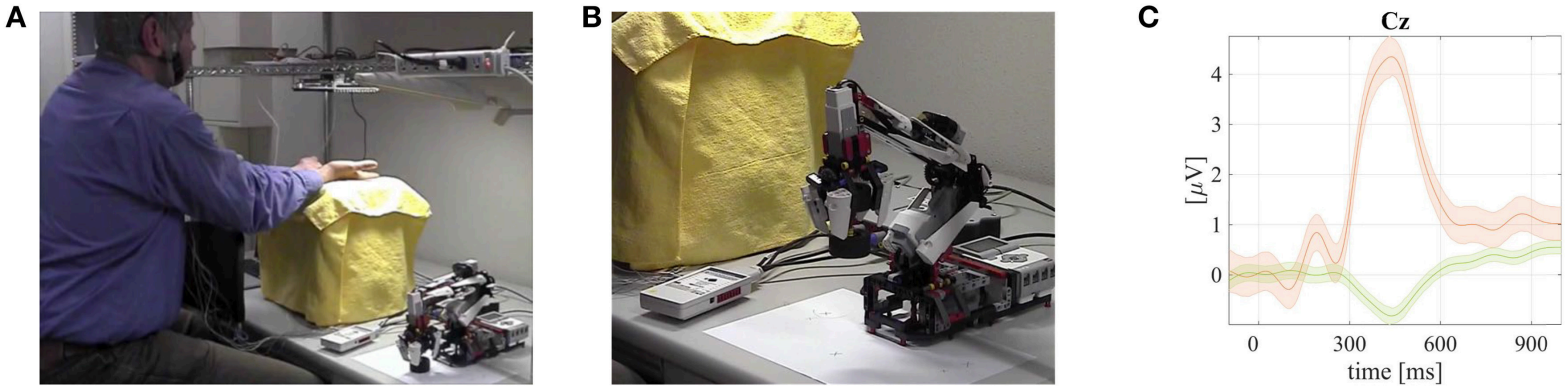
**Screenshots from a video available online^**4**^ that demonstrates the RautdBCI–based LEGO robotic prosthetic arm model control and grand mean averaged tactile ERPs with P300 responses visualized**. Published with permission of the depicted user. **(A)** A user wering EEG cap and placing his hands under AUTD array. **(B)** The LEGO robotic prosthetic hand model during RautdBCI control experiment. **(C)** Grand mean averaged ERP of the rare targets with P300 (orange line) and non–targets (green) together with standard error intervals from RautdBCI experiments.

**Figure 5:**
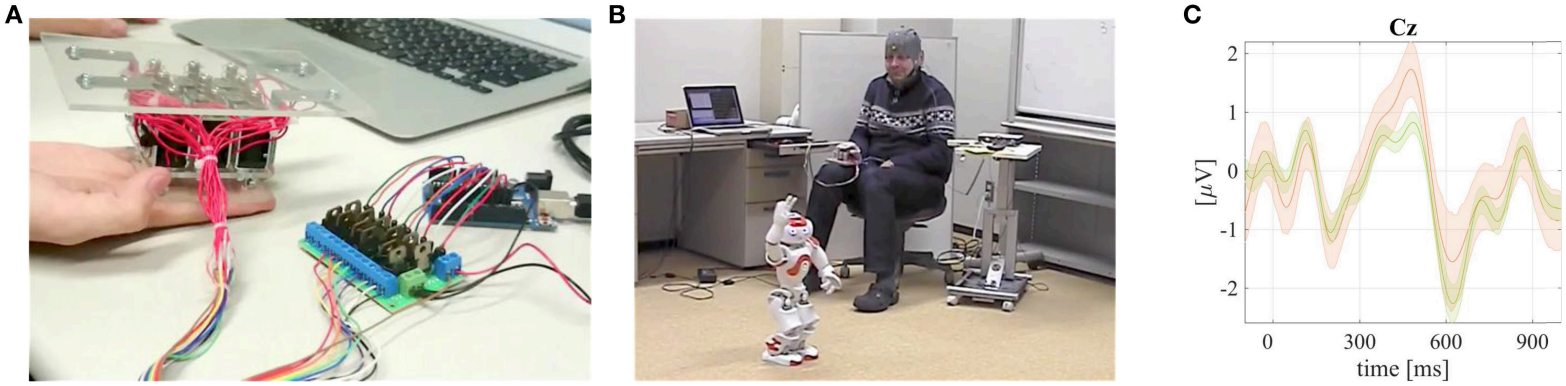
**Screenshots from a video available online^**5**^ that demonstrates the robotic tactile pin–pressure BCI (RtppBCI) in application to the NAO humanoid robot control and grand mean averaged tactile ERPs with P300 responses visualized**. Published with permission of the depicted user. **(A)** The pin–pressure device placed on a user palm and used in the RtppBCI experiments. **(B)** User controlling the humanoid NAO robot with the RtppBCI. **(C)** rand mean averaged ERP of the rare targets with P300 (orange line) and non-targets (green) together with standard error intervals from RtppBCI experiments.

**Figure 6:**
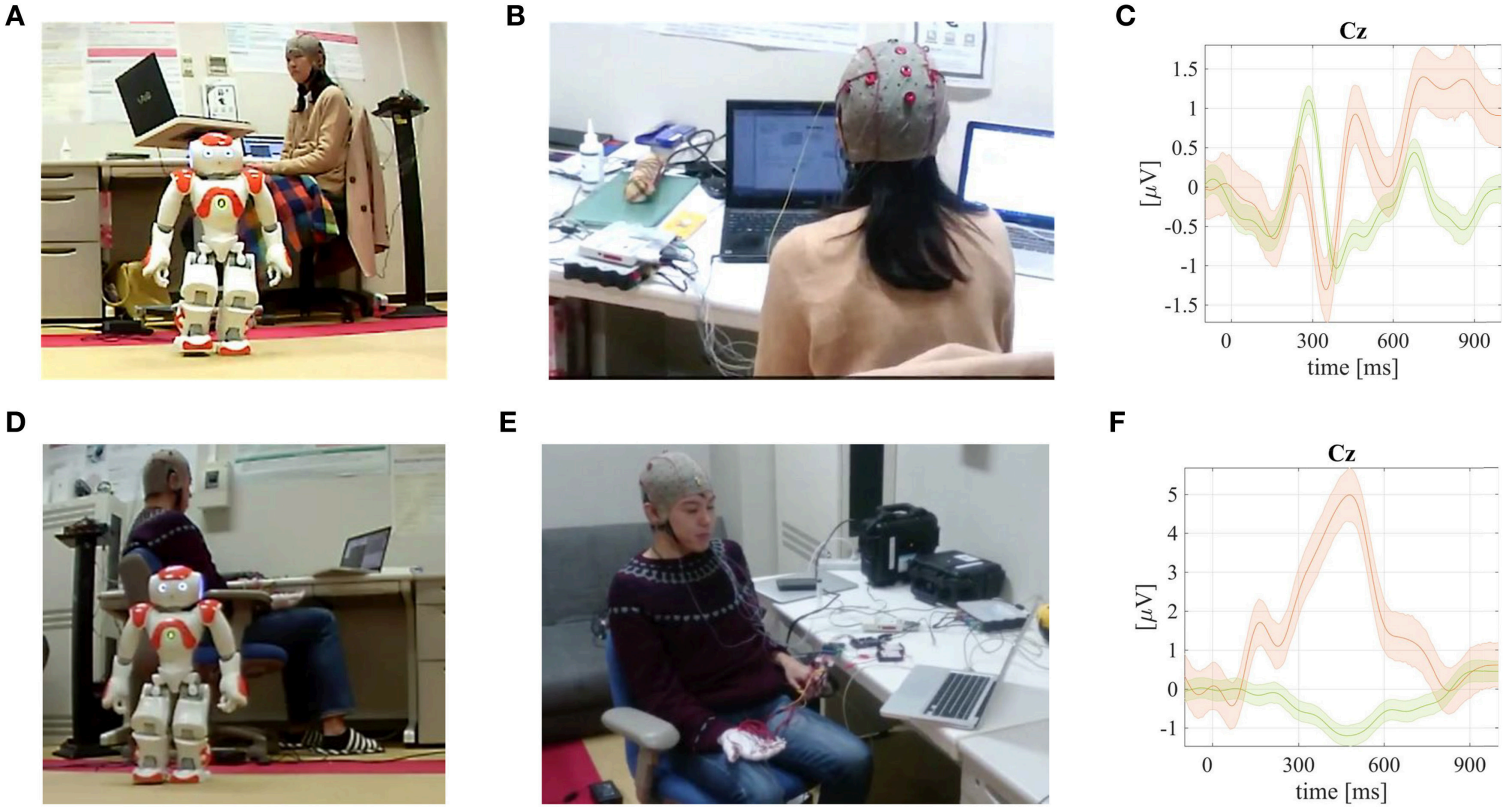
**Screenshots from a video available online^**6**^ that demonstrates robotic spatial auditory (RsaBCI) and tactile–glove (RtgBCI) BCIs for humanoid robot NAO control**. Grand mean averaged ERPs with P300 responses visualized are also depicted for the both paradigms. Published with permission of the depicted users. **(A)** An user during the RsaBCI–based humanoid NAO robot control. **(B)** An user head with EEG cap and small in–ear headphones during the RsaBCI–based humanoid NAO robot control experiments. **(C)** Grand mean averaged ERP of the rare targets with P300 (orange line) and non–targets (green) together with standard error intervals from RsaBCI experiments. **(D)** An user during the RtgBCI–based humanoid NAO robot control. **(E)** An user head with EEG cap and a hand wearing a glove with vibrotactile transducers used in the RtgBCI–based humanoid robot NAO control experiments. **(F)** Grand mean averaged ERP of the rare targets with P300 (orange line) and non–targets (green) together with standard error intervals from RtgBCI experiments.

**Figure 7:**
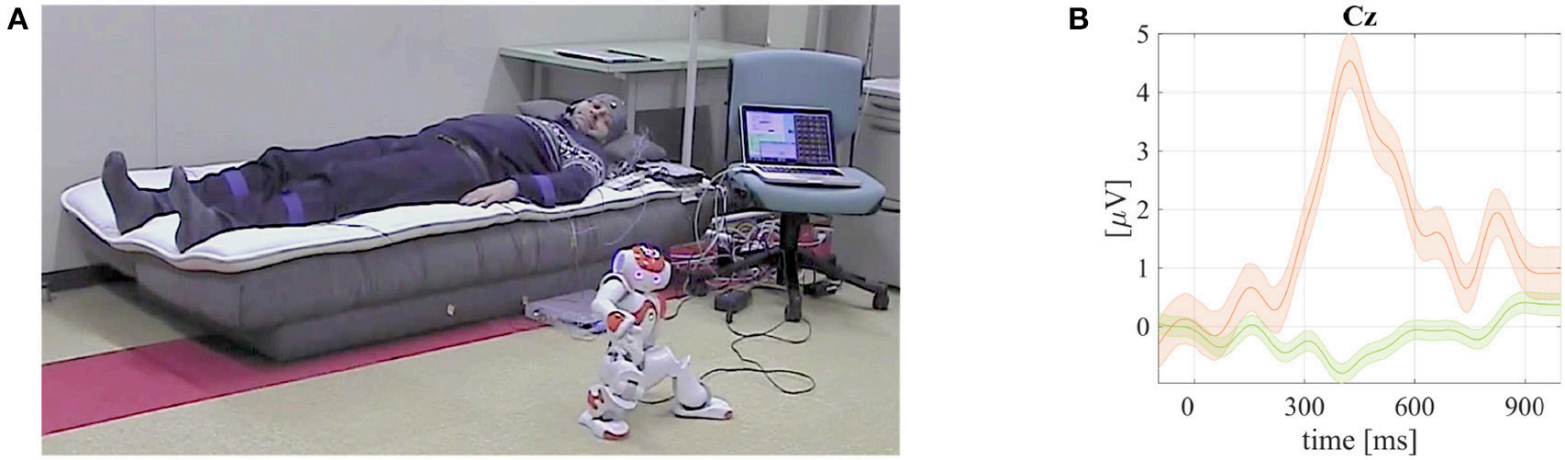
**A screenshot from a video available online^**7**^ that demonstrates the robotic full–body tactile BCI (RfbtBCI) humanoid robot NAO control and grand mean averaged tactile ERPs with P300 responses visualized**. Published with permission of the depicted user. **(A)** A RfbtBCI user lying on a mattress with vibrotactile transducers embedded and controlling the humanoid robot NAO. **(B)** Grand mean averaged ERP of the rare targets with P300 (orange line) and non–targets (green) together with standard error intervals from RfbtBCI experiments.

The P300 responses are usually discriminated in BCI application using a stepwise linear discriminant analysis (SWLDA) classifier method, which is an extension of the classical linear discrimination technique proposed by Krusienski et al. (2006). The SWLDA is broadly applied in many realtime BCI applications (Renard et al., 2010; Schalk and Mellinger, 2010; Stocks, 2011). A problem of event related potentials (ERPs) classification is related to very noisy and transient EEG brainwave discrimination during online experiments. In order to deal with the problem, Barachant et al. (2012) proposed to specify a generic model for the observed data. Let's assume that **x**(*t*) ∈ ℝ^3*N*^ be a zero–mean ERP data sample captured from *N* EEG electrodes, at a discrete time sample *t*, concatenated with averaged matrices of rare targets and non-targets (*N*-channels each) of the oddball BCI paradigm (Wolpaw and Wolpaw, 2012). The trick with adding the averaged references of the rare targets and non-targets is to compensate for a lack of a spatial variability of the ERP responses. Let also ${\text{x}}_{k,i}\in {\mathbb{R}}^{3N}$ be a single ERP, together with reference averages from a training session number *i*, which is a member of rare targets (carrying the P300 responses) or non-targets (no P300). The rare targets carry EEG positive deflections usually within 300–600 ms interval latencies (see Figures 1D,E, 2B, 3C, 4C, 5C, 6C,F, 7B with various sensory modality–based P300 responses from robotic and VR BCI experiments discussed in this paper). Each captured ERP thus belongs to *k* ∈ {1, 2} class and it contains *M* samples. In this study all ERPs from the previously developed by the author and his students robotic as well as VR BCI projects (Mori et al., 2013a; Hamada et al., 2014; Kodama et al., 2014, 2016; Neto et al., 2014; Nakaizumi et al., 2015a; Rutkowski and Mori, 2015; Rutkowski and Shinoda, 2015; Rutkowski et al., 2015b; Shimizu et al., 2015c; Yajima et al., 2015) have been standardized for an easy comparison within latencies of 0–1000 ms. Within an information geometry-based approach proposed by Barachant et al. (2012), each ERP record is assumed to have a zero mean. With the above assumption, a single trial covariance matrix **x**_*k, i*_, which represents ERP from a class *k*, together with the averaged training references, is given as,

(1) $$\begin{array}{l} {\text{C}}_{k,i}=\frac{1}{M-1}{\text{x}}_{k,i}{\text{x}}_{k,i}^{T}, \end{array}$$

where *M* stands for a number of samples in each event **x**_*k, i*_. With an additional assumption of the ERPs being from multivariate Gaussian distributions, a covariance matrix shall be the only unique parameter for each target or non-target response classes. Barachant et al. (2012) proposed a classification algorithm that employs the covariance matrices as input features. In this paper, the same approach is utilized since the covariance matrices convey a satisfactory discriminable information of the monitored EEG brainwave responses. A classification step is usually defined by assigning to an unlabeled ERP an existing and predicted class (rare target vs. non-target responses), which is obtained from the covariance matrix features, **C**_*k, i*_ calculated as in Equation (1), of the input EEG brainwave channels. Barachant et al. (2012) and Congedo et al. (2013) proposed a very natural for information geometry–based (especially the Riemannian geometry) derived features classification algorithm, which is based on fining a minimum distance of the newly acquired ERPs, within the realtime monitored EEG, from the class–representing mean covariance matrices. A minimum distance to mean (MDM) classifier (Barachant et al., 2012; Congedo et al., 2013) meets the above criterium and it has been reviewed in this paper.

During a classifier training phase, geometric mean covariance matrices **C**_*k*_ representing each target or non-target ERP classes *k* are computed using a training phase dataset. Next, in testing steps the remaining datasets are evaluated for the BCI accuracy improvement analysis with the proposed MDM classifier, and in comparison with the classical and perviously used SWLDA technique. The covariance matrices are symmetric and positive definite (Barachant et al., 2012). This implies that they can be diagonalized by a rotation and they have also all positive eigenvalues. In order to compute a distance of a newly arriving ERP trial sample from the above mentioned class-characterizing mean covariance matrix **C**_*k*_ an appropriate metric, allowing a simple discrimination, is employed. A point on a Riemannian manifold represents the symmetric positive definite matrix (Barachant et al., 2012; Barachant and Congedo, 2014; Yger et al., 2016).

The Riemannian distance between two covariance matrices **C**_*i*_ and **C**_*j*_ is defined as follows (Barachant et al., 2012; Amari, 2016),

(2) $$\begin{array}{l} \delta_{R}=||ln\left({\text{C}}_{i}^{-1}{\text{C}}_{j}\right)|{|}_{F}=\sqrt{\underset{n}{\sum }{\left[ln\left(w_{n}\right)\right]}^{2}}, \end{array}$$

where the symbol ||·|| denotes a Frobenius norm and *w*_1_, …, *w*_*n*_ the eigenvalues of ${\text{C}}_{i}^{-1}{\text{C}}_{j}$, respectively. The geometric mean, using the Riemannian distance defined in Equation (2), of *J* covariance matrices is computed as follows (Barachant and Congedo, 2014),

(3) $$\begin{array}{l} 𝔊\left({\text{C}}_{1},\cdots \;,{\text{C}}_{J}\right)=\arg\underset{\text{C}}{\min}\overset{J}{\underset{j=1}{\sum }}\delta_{R}^{2}\left(\text{C},{\text{C}}_{j}\right). \end{array}$$

The class representing mean covariance matrix **C**_*k*_ denoted by corresponding labels *l*_*i*_ ∈ {1, 2, …, *k*} and calculated within the MDM classifier training process, is thus obtained as,

(4) $$\begin{array}{l} {\bar{\text{C}}}_{k}=𝔊\left({\text{C}}_{i}|l_{i}=k\right). \end{array}$$

A the classification step, originally proposed by Barachant and Congedo (2014), a new ERP trial is assigned a label by finding the minimum distance among the class-representing means as follows,

(5) $$\begin{array}{l} \hat{l}=\underset{k}{\arg\min}\delta_{R}\left({\bar{C}}_{k},C\right). \end{array}$$

The above shortly introduced classification method employing the information geometry principles to EEG brainwaves, constitutes a very generic approach allowing for a minimization of the ERP averaging number and resulting with BCI classification accuracy boosting. The MDM classifier is a novel and superior method, comparing to the classical SWLDA as shown in following sections reviewing robotic and VR BCIs applications.

## 3. Results

In this section, the previously developed virtual reality (VR) (Neto et al., 2014) and robotic (Mori et al., 2013a; Hamada et al., 2014; Kodama et al., 2014, 2016; Nakaizumi et al., 2015a; Rutkowski and Mori, 2015; Rutkowski and Shinoda, 2015; Rutkowski et al., 2015b; Shimizu et al., 2015c; Yajima et al., 2015) BCIs are reviewed together with new results reporting BCI accuracy boosting in offline analysis set-up using the proposed information geometry MDM classifier approach introduced in Section 2.1.

### 3.1. VR tactile bone-conduction auditory (VRtbaBCI) and tactile pin-push (VRtppBCI) BCIs

A virtual reality (VR) study has been conducted at the BCI-lab research group and it covered two BCI prototypes tested using online (realtime) applications (Neto et al., 2014), as also depicted in Figure 1. The first VR BCI (vrBCI) type has been developed for those paralyzed or locked-in syndrome (LIS) (Plum and Posner, 1966) patients, who could not see visual stimuli or hear air-pressure-conducted sounds due the advanced stages of their diseases (bad eyesight or blocked external ear canals) (Gelinas, 2007; Rutkowski and Mori, 2015). Our group developed a tactile and bone-conduction auditory brainwave evoked response-based BCI prototype (VRtbaBCI) using vibrotacile transducers attached to a user head and operating in acoustic frequencies (Mori et al., 2013d; Rutkowski and Mori, 2015), as shown also in Figure 1A. The second VR BCI prototype, discussed in this section, has been developed for a rapid tactile stimulus presentation based on a small pin-push pattern (VRtppBCI) applied to user fingers using a small matrix of nine solenoids controlled by a personal computer (Shimizu et al., 2014, 2015c), as also depicted in Figure 1C. The experimental VR scene was developed within the Unity3D gaming engine. A chosen virtual agent resembled a young gentleman walking in a an open space with cubic obstacles as shown in Figure 1B. The virtual agent was able to walk freely and it was controlled within a two dimensional space using commands from the vrBCI application. The agent could execute the following commands: *walk-straight, return, walk-left, walk-right*, or to *stop*. Within the virtual space (see Figure 1B) four obstacle cubes were placed for visual reference and in order to define a walking path for the user to follow. The vrBCI users executed several walking path scenarios around the cubes.

During the VR BCI experiments, evaluating the VRtbaBCI and VRtppBCI paradigms, EEG signals were recorded by the g.USBamp EEG bio-amplifier using the g.GAMMAbox with attached eight active electrodes g.LADYbird (g.tec Medical Instruments GmbH, Austria). The EEG electrodes were placed on the 10/10 extended international system head locations: *Cz, Pz, P3, P4, C3, C4, CP5*, as well as *CP6*. A grounding electrode was connected to *FPz* position and a reference, of the unipolar recording montage, to a left earlobe. We did not observe any electromagnetic interference on EEG caused by the vibrotactile exciters (electric coils), which operated in higher acoustic frequencies (above 100 Hz), as well as from solenoids placed on the user hand using a vinyl isolating glove.

The recorded EEG signals were processed in realtime by an in-house expanded BCI experimental environment BCI2000 (Schalk and Mellinger, 2010; Matsumoto et al., 2013). Experimental stimuli in the both discussed VRtbaBCI and VRtppBCI paradigms were presented with an inter-stimulus-interval (ISI) of 150 ms. Each single stimulus duration was set to 100 ms length. For the online P300 responses classification we used a stepwise linear discriminant analysis (SWLDA) classifier (Krusienski et al., 2006), while in offline EEG post-processing the MDM has been applied as introduced in Section 2.1. The bone-conduction auditory and tactile stimuli were generated by vibrotactile transducers and solenoids controlled by ARDUINO UNO micro-controller board managed from the visual multimedia programming environment MAX (Cycling'74, USA). The BCI commands were sent also from the same MAX environment using the UDP protocol to the virtual reality agent programmed in the Unity3D, as an internet-of-things (IoT) scenario set-up. A pre-defined walking path within the VR environment was explained, before each of the experiments, to the BCI user. The user was requested to follow the path. The BCI controlled commands were *walk up, down, left, right, or stop*, as can be seen in an online video1. Averaged BCI accuracy results for head tactile bone-conduction auditory BCI using limited number of averaged ERPs [smaller in comparison to the previous report by Neto et al. (2014)] are summarized in Table 1 and depicted in form of grand means in Figure 1D. Similarly, for the case of VRtppBCI paradigm, the BCI accuracy results are summarized in Table 1 and Figure 1E, respectively. For the both above cases introduction of the MDM classifier for a limited number of averaged ERPs allowed for a significant boost of BCI accuracy results.

**Table 1:** **The VRtbcaBCI and VRtppBCI classification accuracies obtained with the information geometry–based MDM classifier versus the standard SWLDA method together with grand mean difference significance ***p***-values obtained from pairwise rank–sum tests**.

**Classifier type**	**BCI accuracy depending on a number of averaged ERPs**				
	**1**	**2**	**3**	**4**	**5**
**VRtbcaBCI**					
MDM (%)	55.4±7.9	80.9±9.9	95.7±4.9	99.6±2.0	100.00±0.0
SWLDA (%)	22.1±18.3	24.1±18.8	23.9±16.3	22.1±16.3	26.2±15.0
Significance	*p* ≪ 0.001	*p* ≪ 0.001	*p* ≪ 0.001	*p* ≪ 0.001	*p* ≪ 0.001
**VRtppBCI**					
MDM (%)	58.5±12.0	74.2±12.6	68.7±18.3	54.2±28.6	84.1±3.3
SWLDA (%)	22.4±17.2	23.5±20.3	25.9±22.1	23.5±20.3	23.5±21.5
Significance	*p* ≪ 0.001	*p* ≪ 0.001	*p* ≪ 0.001	*p* < 0.01	*p* ≪ 0.001

The above discussed VR environment study results, in the online BCI experimental set-up, confirmed the proposed tactile and bone-conduction auditory modalities-based agent control paradigm validities. Even if the vrBCI concept may still need improvements, we have shown that the proposed paradigm could lead to development of efficient and comfortable virtual reality applications.

### 3.2. Robotic tactile chest BCI (RtcBCI) for a LEGO vehicular robot control

The first direct brain-robotics project developed at the BCI-lab research group created a multi-command robotic tactile chest BCI (RtcBCI) for a small vehicular LEGO robot control (Mori et al., 2013a), as depicted in Figure 2. This research project was an improvement of a previously reported finger stimulus tactile BCI developed also by the BCI–lab research group (Mori et al., 2013b). The direct brain–robotics interface was based on the P300 response (Donchin and Coles, 1988) classification in a tactile sensory modality (Brouwer and Van Erp, 2010). The ERPs were evoked by tactile stimuli generated by vibrotactile transducers attached to five chest positions (Mori et al., 2013a). In the reported study five male volunteers participated with a mean age of 26.6 ± 9.5 years. Tactile stimuli were delivered as sinusoidal waves generated by a portable computer with MAX software (Cycling'74, USA), via five channel outputs of an external digital-to-analog signal converter RME Fireface UCX, (RME, Germany) coupled with the two acoustic YAMAHA P4050 power amplifiers (YAMAHA, Japan). The stimuli were delivered to the user chest locations via the tactile transducers HiWave HIAX25C10-8/HS operating at 200 Hz to match their resonance frequencies. The tactile pulses were designed to stimulate the *Pacini endings* (fast-adapting type II afferent type tactile sensory innervation receptors) which are the large receptive field mechanoreceptors in deeper layers of human skin (Johansson and Flanagan, 2009). Instructions during a training session were presented visually by means of the BCI2000 program (Schalk and Mellinger, 2010; Matsumoto et al., 2013) with numbers 1, 2, …5 representing robot movement directions (#1: move left at −90°; #2: straight-left at −45°; #3: straight at 0°; #4: straight–right at 45°; and #5: right at 90°) and communicated via vibrotactile transducers attached to the user chest (Mori et al., 2013a).

During the online EEG experiment, brainwaves were captured using a bio-amplifier system g.USBamp (g.tec Medical Instruments GmbH, Austria). Sixteen active electrodes were attached to the 10/10 international system head locations (Jurcak et al., 2007) as follows: *Cz, Pz, P3, P4, C3, C4, CP5, CP6, P1, P2, POz, C1, C2, FC1, FC2*, and *FCz*. A ground electrode was connected to the *FPz* position, while a reference to a left earlobe. An electromagnetic interference from the vibrotactile transducer was not observed since it operated in higher frequencies comparing to the EEG spectrum (above 100 Hz). The captured EEG signals were processed online using an in-house extended BCI2000 application (Schalk and Mellinger, 2010; Matsumoto et al., 2013). The LEGO robot control application demonstrated a feasibility of the five-commands and chest locations based RtcBCI paradigm. The RtcBCI was tested with five “body-able” users. The RtcBCI allowed for a real-time operation of a robotic small vehicle as visualized in Figure 2A or in an online video available online.^1^ The experimental BCI accuracy results using SWLDA and MDM classifiers (a chance level of 20.0%) are summarized in Table 2. Also in this case the offline MDM classifier application resulted with significantly better results, as tested with a pairwise rank-sum Wilcoxon test. The obtained grand mean averaged rare target (carrying P300 responses) and non-target ERPs are depicted in Figure 2B.

**Table 2:** **The RtcBCI classification accuracies (a chance level of 20.0%) obtained with the information geometry–based MDM classifier versus the standard SWLDA method together with grand mean difference significance ***p***-values obtained from pairwise rank–sum tests**.

**Classifier type**	**BCI accuracy depending on a number of averaged ERPs**				
	**1**	**2**	**3**	**4**	**5**
MDM (%)	61.1±12.5	99.6±0.8	100.00±0.0	100.00±0.0	100.00±0.0
SWLDA (%)	23.0±23.2	22.0±23.2	26.2±19.6	26.8±20.8	24.8±23.0
Significance	*p* ≪ 0.001	*p* ≪ 0.001	*p* ≪ 0.001	*p* ≪ 0.001	*p* ≪ 0.001

### 3.3. Robotic body–tactile BCI (RbtBCI) for a LEGO prosthetic hand model control

The second robotic project, conducted at the BCI-lab research group, was carried out by applying vibration stimuli to the user back (see Figure 3A). Such experimental set-up allowed for stimulation of places at larger distances on a user body for a robotic body-tactile BCI (RbtBCI) paradigm (Kodama et al., 2014, 2015). The RbtBCI paradigm has been tested with a LEGO robotic prosthetic hand model depicted in Figure 3B. In the experiments the user lied down on the vibrotactile stimulating pad (Comfort Research, USA). The user interacted with stimulus patterns delivered to the whole back of the body in an oddball-style (Wolpaw and Wolpaw, 2012) paradigm, as illustrated in Figure 3A. In the reported experiments seven healthy BCI–naive users participated. An average age was of 25.0 ± 7.8 years. In the RbtBCI online experiments, the EEG signals were acquired with a bio-signal amplifier g.USBamp (g.tec Medical Instruments GmbH, Austria). We used sixteen active EEG electrodes (*Cz,P3, P4, Pz, C3, C4, CP5, CP6, P1, P2, POz, C1, C2, FC1, FC2*, and *FCz*). The EEG signals were captured and classified during online RbtBCI experiments by in-house modified BCI2000 software. The EEG sampling frequency was set to 512 Hz in all trials. The vibrotactile spatial pattern stimuli in the two experimental settings were generated using the MAX visual programing environment (Cycling'74, USA) with a program developed by our team. The RbtBCI experimental accuracy results using SWLDA and MDM classifiers are summarized in Table 3. In this case also the offline MDM classifier application resulted with significantly better results, as tested with a pairwise rank-sum Wilcoxon test. The obtained grand mean averaged rare target (carrying P300 responses) and non-target ERPs are depicted in Figure 3C. The chance level was at 16.7%.

**Table 3:** **The RbtBCI classification accuracies (a chance level was at 16.7%) obtained with the information geometry–based MDM classifier vs. the standard SWLDA method together with grand mean difference significance ***p***-values obtained from pairwise rank–sum tests**.

**Classifier type**	**BCI accuracy depending on a number of averaged ERPs**				
	**1**	**2**	**3**	**4**	**5**
MDM (%)	51.4±9.9	68.8±5.4	94.7±8.3	99.8±1.3	100.0±0.0
SWLDA (%)	22.2±9.9	17.2±5.4	26.3±8.3	23.2±1.3	22.7±0.0
Significance	*p* ≪ 0.001	*p* ≪ 0.001	*p* ≪ 0.001	*p* ≪ 0.001	*p* ≪ 0.001

### 3.4. Robotic airborne ultrasonic tactile display–based BCI (RautdBCI) for a LEGO prosthetic arm model control

The airborne ultrasonic tactile display (AUTD) based BCI received The BCI Annual Research Award 2014 and it allowed for a creation of several very interesting applications (Hamada et al., 2014, 2015; Rutkowski et al., 2014, 2015a; Rutkowski and Shinoda, 2015) including a robotic AUTD paradigm (RautdBCI) applied to a LEGO prosthetic hand model control described in this section (see Figure 4). The AUTD stimulus generator in the RautdBCI experiments delivered tactile and contactless stimuli to the user skin only via air pressure modulation using a technique of beam-forming-based focused ultrasound (Iwamoto et al., 2008; Mori et al., 2012, 2013a,b,c,d; Hamada, 2014). The AUTD stimulation effect was achieved due to a generated ultrasonic radiation static force, which was produced by an intense sound pressure amplitude. The effect was a result of a nonlinear acoustic phenomenon (Iwamoto et al., 2008). The above mentioned radiation pressure allowed for a deformation of a skin surface of hand fingers and palms, creating thus a virtual touch sensation (“a contactless somatosensory stimulation”). Next, the modulated radiation air-pressure brought a sensation of the tactile vibration, which was similar to the mechanical equivalent delivered by the classical vibrotactile transducers usually attached to the user skin (Yajima et al., 2014, 2015). The AUTD device (Iwamoto et al., 2008; Hamada, 2014) used in our study complied with ultrasonic medical standards. The permitted skin absorption levels were not exceed (approximately 40 times below the permitted limits). The vibrotactile sensation used in the study was set to 50 Hz (Hamada, 2014) in order to match frequency tuning characteristics of tactile skin mechanoreceptors (Iwamoto et al., 2008; Mori et al., 2012). Also the above frequency was aligned with the notch filter that EEG amplifiers use for power line interference rejection. Thirteen male volunteer BCI users participated in the experiments. An average age of the users was of 28.54 ± 7.96 years.

The user brainwaves were acquired using the bio-signal amplifier g.USBamp (g.tec Medical Instruments GmbH, Austria). The electrodes were applied to the *Pz, Cz, P3, P4, C3, C4, CP5, CP6, P1, P2, POz, FC1, FC2, C1, C2*, and *FCz* as in the 10/10 extended international system head locations. A ground electrode was applied to the *FPz* and a reference to a left earlobe, respectively. No electromagnetic interference was observed from the AUTD.

The users were given an instruction to copy-spell six digits sequences representing the stimulated places on their fingers and palms. The digits were mapped to robotic prosthetic hand model control (six pre-programmed movements allowing for simple picking and moving small objects) as shown in Figures 4A,B. The RautdBCI experimental accuracy results using SWLDA and MDM classifiers are summarized in Table 4. The offline MDM classifier application applied to the RautdBCI resulted, also in this case, with significantly better results, as tested with a pairwise rank-sum Wilcoxon test. The obtained grand mean averaged rare target (carrying P300 responses) and non-target ERPs are depicted in Figure 4C. The chance level was at 16.7%. A video demonstrating a fully successful robot control is available online^**4**^.

**Table 4:** **The RautdBCI classification accuracies (a chance level at 16.7%) obtained with the information geometry–based MDM classifier vs. the standard SWLDA method together with grand mean difference significance ***p***-values obtained from pairwise rank–sum tests**.

**Classifier type**	**BCI accuracy depending on a number of averaged ERPs**				
	**1**	**2**	**3**	**4**	**5**
MDM (%)	54.7±14.3	71.7±3.2	91.4±10.9	98.3±3.8	99.8±1.5
SWLDA (%)	20.6±19.4	23.6±19.5	27.1±20.6	26.6±20.8	30.1±23.1
Significance	*p* ≪ 0.001	*p* ≪ 0.001	*p* ≪ 0.001	*p* ≪ 0.001	*p* ≪ 0.001

### 3.5. Hand tactile pin-pressure BCI (RtppBCI) for the humanoid robot NAO control

In the fourth robotic project conducted by the BCI-lab research group also a tactile stimulus generator was utilized. A tactile pin-pressure generator used in the study was composed of nine solenoids arranged in 3 × 3 point matrix (Shimizu et al., 2014). Six stimulus linear patterns were composed of the tactile pressure pins and they were delivered in random order to the user fingers (Rutkowski et al., 2015b,c; Shimizu et al., 2015a). The robotic tactile pin-pressure BCI (RtppBCI) is presented in Figures 5A,B. Three of the linear patterns were horizontal arrangements ordered from the top to the bottom of user fingers. The remaining three patterns were vertical in the left to right order. The solenoid generated linear pressure patterns were at each time 100 ms long. The decoded from EEG brainwave BCI commands were sent next to the robot via the UDP network protocol packets using a wireless connection (the IoT scenario). The humanoid robot NAO executed the pre-programmed movements (user intended commands) as a result of the successfully classified P300 responses to stimuli delivered to the hand of the user (see Figure 5). EEG signals were acquired, during online BCI experiments from eight active EEG electrodes *Cz, P3, P4, Cpz, C3, C4, CP5*, and *CP6*. A reference electrode was connected as an ear-clip to a left earlobe. A ground electrode was attached on a forehead at *FPz* location, similarly as in a study by Shimizu et al. (2015c). The user brainwaves were acquired using the bio-signal amplifier g.USBamp (g.tec Medical Instruments GmbH, Austria). The user wore on polyethylene glove in order to limit a possible electric interference leaking from the tactile generators. The users were also asked to possibly limit body and eye movements in order avoid EMG interference. The EEG signals were acquired and processed by our in-house expanded BCI2000 application (Schalk and Mellinger, 2010; Matsumoto et al., 2013). We used in online experiments, similarly as in previous sections, the SWLDA classifier (Krusienski et al., 2006). The EEG sampling rate was of 256 Hz. The ISI was set at 400 ms length and each stimulus duration of 100 ms, respectively. The humanoid NAO robot was controlled using six pre-programmed commands, which were decoded from user EEG and classified by the developed RtppBCI. The commands were transmitted from the RtppBCI to the robot in form of numbers representing the six movement commands. Ten male users participated in the study with an average age of 24.8 ± 3.8 years. The RtppBCI experimental accuracy results using the classical SWLDA and the proposed MDM classifiers are summarized in Table 5. The offline MDM classifier application applied to the RtppBCI concluded, also in this case, with significantly higher results, as tested with a pairwise rank-sum Wilcoxon test. The obtained grand mean averaged rare target (carrying P300 responses) and non-target ERPs are depicted in Figure 5C. The chance level was at 16.7%. A video demonstrating a fully successful robot control is available online^**5**^.

**Table 5:** **The RtppBCI classification accuracies (a chance level at 16.7%) obtained with the information geometry–based MDM classifier vs. the standard SWLDA method together with grand mean difference significance ***p***-values obtained from pairwise rank–sum tests**.

**Classifier type**	**BCI accuracy depending on a number of averaged ERPs**				
	**1**	**2**	**3**	**4**	**5**
MDM (%)	50.8±17.8	71.5±1.8	71.7±2.6	72.7±4.2	100.0±0.0
SWLDA (%)	16.7±20.7	22.0±17.5	24.0±22.7	22.0±19.9	20.0±21.1
Significance	*p* < 0.001	*p* ≪ 0.001	*p* ≪ 0.001	*p* ≪ 0.001	*p* ≪ 0.001

### 3.6. Robotic spatial auditory BCI (RsaBCI) for the humanoid robot NAO control

In the fifth robotic project conducted by the BCI–lab research group, five spatial sound (auditory) stimuli were used in an oddball paradigm (Nakaizumi et al., 2014, 2015a,b) as shown in Figures 6A,B. Five Japanese vowels were positioned spatially on a virtual horizontal acoustic plane reproduced using headphones with head-related impulse responses (HRIRs) by Nakaizumi et al. (2014). The experimental azimuth locations were left side at −80° and −40°; center at 0°; and right at 40° and 80°, for the Japanese kana vowels of *a, i, u, e*, and *o*, respectively. A synthetic computer voice was used in the experiments. The online EEG experiments were conducted to investigate P300 response validity for a robotic spatial auditory BCI (RsaBCI) thought-based control (see Figure 6C with the grand mean averaged auditory ERPs). The brain signals were recorded by a bio-signal amplifier g.USBamp (g.tec Medical Instruments GmbH, Austria). The EEG signals were acquired using sixteen active electrodes attached to the following head position *Cz, P3, P4, Pz„ Cp5, Cp6, P1, P2, Poz, C1, C2, FC1, FC2*, and *FCz*. The ground electrode was connected on the forehead at the *FPz* and the reference on the left earlobe. Our laboratory in-house expanded BCI2000 (Schalk and Mellinger, 2010; Matsumoto et al., 2013) application together with MAX environment (Cycling'74, USA) were used for the online spatial auditory BCI (saBCI) experiments to present stimuli and display online classification results. A single stimulus duration was set to 150 ms. The inter-stimulus-interval (ISI) was set to 150 ms. The EEG sampling rate was of 512 Hz. Five female users participated in the study with an average age of 21.6 ± 0.5 years. The RsaBCI experimental accuracy results using the classical SWLDA and the proposed MDM classifiers are summarized in Table 6. The offline MDM classifier application applied to the RsaBCI also in this case resulted with significantly higher outcomes, as tested with a pairwise rank-sum Wilcoxon test. The obtained grand mean averaged rare target (with the P300 responses) and non-target ERPs are depicted in Figure 6F. The chance level was at 20.0%. A video demonstrating a fully successful robot control is available online^**6**^.

**Table 6:** **The RsaBCI classification accuracies (a chance level at 20.0%) obtained with the information geometry–based MDM classifier vs. the standard SWLDA method together with grand mean difference significance ***p***-values obtained from pairwise rank–sum tests**.

**Classifier type**	**BCI accuracy depending on a number of averaged ERPs**				
	**1**	**2**	**3**	**4**	**5**
MDM (%)	55.22±5.6	59.0±4.8	98.0±3.4	97.3±3.0	99.2±1.1
SWLDA (%)	28.3±22.6	28.9±19.4	26.1±22.8	32.8±27.5	32.2±26.7
Significance	*p* ≪ 0.001	*p* ≪ 0.001	*p* ≪ 0.001	*p* ≪ 0.001	*p* ≪ 0.001

### 3.7. Robotic tactile–glove BCI (RtgBCI) for the humanoid robot NAO control

In the sixth robotic project conducted by the BCI–lab research group, a somatosensory stimulator in a form of a “tactile–glove” was employed (Yajima et al., 2014, 2015) in order to create a robotic tactile-glove BCI (RtgBCI) as presented in Figures 6D,E. Nine vibrotactile exciters were attached to user fingers. Each finger, except thumb, had two vibrotactile exciters attached. Similarly as in previously discussed BCI paradigms, intentionally modulated tactile P300 responses were translated to the humanoid NAO robot control commands as shown in Figure 6. The user brainwaves were acquired using the bio-signal amplifier g.USBamp (g.tec Medical Instruments GmbH, Austria). The EEG signals were recorded from eight active electrodes, which were attached to the following head positions of *Cz, P3, P4, C3, C4, CPz, Cp5*, and *CP6*, respectively. The ground electrode was set on a forehead (*FPz*), and a reference on a left earlobe. The extended BCI2000 software (Schalk and Mellinger, 2010; Matsumoto et al., 2013) together with MAX environment (Cycling'74, USA) were used in the experiments. The acquired and filtered brain signals were next segmented as well as finally classified online, after a preliminary training, by the SWLDA classifier, also in this scenario. Five male users participated in the study with an average age of 26.6 ± 9.5 years. The RtgBCI experimental accuracy results using the classical SWLDA and the proposed MDM classifiers are summarized in Table 7. The offline MDM classifier application applied to the RtgBCI, as well as in this case, resulted with significantly higher outcomes, as tested with a pairwise rank-sum Wilcoxon test. The obtained grand mean averaged rare target (with the P300 responses) and non-target ERPs are depicted in Figure 6C. The chance level was at 20.0%. A demonstration video with a realtime humanoid robot NAO control as available online^**6**^.

**Table 7:** **The RtgBCI classification accuracies (a chance level at 20.0%) obtained with the information geometry–based MDM classifier vs. the standard SWLDA method together with grand mean difference significance ***p***-values obtained from pairwise rank–sum tests**.

**Classifier type**	**BCI accuracy depending on a number of averaged ERPs**				
	**1**	**2**	**3**	**4**	**5**
MDM (%)	55.6±8.2	70.8±2.4	73.1±0.7	89.1±10.5	100.0±0.0
SWLDA (%)	25.0±25.9	28.6±21.6	26.8±20.7	26.8±20.7	35.7±23.4
Significance	*p* < 0.001	*p* ≪ 0.001	*p* ≪ 0.001	*p* ≪ 0.001	*p* ≪ 0.001

### 3.8. Robotic full–body tactile stimulation–based BCI (RfbtBCI) for the humanoid robot NAO control

In the final robotic study conducted by the BCI-lab research group, a stimulus-driven tactile BCI have been developed, in which somatosensory stimuli were given to the full body of the user in order to evoke tactile P300 responses (Rutkowski et al., 2015b,c; Shimizu et al., 2015a,b; Kodama et al., 2016) as depicted in Figure 7A. Six spatial tactile stimuli were delivered to various body locations of the users entire back. The classified BCI results were next employed for an intuitive robot control-based application. The robotic control was designed for a paralyzed user who would be in a bedridden condition. An approach has been defined as a robotic full body BCI (RfbtBCI). In the proposed RfbtBCI paradigm, the tactile P300 generating stimuli were applied (Rutkowski et al., 2015b,c; Shimizu et al., 2015a,b; Kodama et al., 2016) to the user back and waist. The user responded mentally only to the identified rare targets as in the classical oddball set-up (Wolpaw and Wolpaw, 2012). The presented approach has been in an active development in mind for clinical conditions and for locked-in syndrome (LIS) bedridden users, although at the current stage of the presented pilot study only healthy participants were tested. For this reason, we developed a tactile stimulus generator applying vibration patterns to a full body of the user's back (Rutkowski et al., 2015b,c; Shimizu et al., 2015a,b; Kodama et al., 2016). Tactile transducers DAYTON TT25-16 were embedded within a mattress in order to generate somatosensory evoked potentials (SEP) with intentionally modulated P300 responses. The tactile stimuli were applied to six distinct areas of the user back and limbs (both arms and legs; waist; and shoulder areas). The NAO humanoid robot was controlled by the RfbtBCI direct brain-robot communication application. Six robot preprogrammed movements were mapped to the RfbtBCI commands (e.g., walk ahead; return; left; or right; sit; and say “goodbye”). The developed direct brain-robot control application is depicted in Figure 7.

The EEG signals were acquired with a bio-signal amplifier g.USBamp (g.tec Medical Instruments GmbH, Austria), and processed using in–house extended BCI2000 environment (Schalk and Mellinger, 2010; Matsumoto et al., 2013). The online P300 responses were classified using the SWLDA method. Eight active EEG g.LADYbird electrodes were attached to eight locations of *Cz, Pz, P3, P4, C3, C4, CP5*, and *CP6*, as in 10/10 international system. The EEG sampling rate was set to 512 Hz. Ten healthy users (five males and five females; mean age of 21.9 ± 1.45) took part in the study. The RfbtBCI experimental accuracy results using the classical SWLDA and the proposed MDM classifiers are summarized in Table 8. The offline MDM classifier application applied to the RfbtBCI, also here, resulted with significantly higher accuracies, as tested with a pairwise rank-sum Wilcoxon test. The obtained grand mean averaged rare target (with the P300 responses) and non-target ERPs are depicted in Figure 7B. The chance level was at 16.7%. This direct brain-robot control project shall be considered as a relatively novel approach. We could successfully realize a full body tactile BCI to bring closer to reality a concept of the direct brain-robot control application (Rutkowski et al., 2015b,c; Shimizu et al., 2015a,b; Kodama et al., 2016). A demonstration video with a user controlling the NAO humanoid robot with the discussed above RfbtBCI paradigm is available online^**7**^.

**Table 8:** **The RfbtBCI classification accuracies (a chance level at 16.7%) obtained with the information geometry–based MDM classifier vs. the standard SWLDA method together with grand mean difference significance ***p***-values obtained from pairwise rank–sum Wilcoxon tests**.

**Classifier type**	**BCI accuracy depending on a number of averaged ERPs**				
	**1**	**2**	**3**	**4**	**5**
MDM (%)	59.8±10.0	71.9±1.2	93.6±9.1	99.1±4.5	99.1±4.5
SWLDA (%)	26.4±18.7	28.8±20.3	32.8±22.0	39.2±23.9	44.0±21.8
Significance	*p* ≪ 0.001	*p* ≪ 0.001	*p* ≪ 0.001	*p* ≪ 0.001	*p* ≪ 0.001

## 4. Discussion

The paper reviewed nine realtime implementations of robotic and VR BCI paradigms developed by the BCI-lab research group. Realtime virtual reality agent and robotic device control scenarios have been explained. The novel information geometry-based MDM method have been introduced, which boosted the VR and robotic BCI accuracies in offline EEG processing applications.

The previously reported VR and robotic BCI paradigms employed the SWLDA classifier that required larger number of averaged ERPs (10–15), which resulted in slower interfacing speeds. The MDM classier introduced in this paper, and compared with the previous SWLDA method, required smaller ERP averaging scenarios (1–5) that allowed for perfect scores achievement for the majority of the tested, and reviewed in this paper, VR and robotic BCIs.

The results obtained with the proposed MDM classifier where significantly better (*p* < 0.001), as tested with pairwise rank-sum Wilcoxon tests, comparing to the classical SWLDA method. The MDM-based classification boosting results were also independent of the presented oddball BCI stimulation modalities (auditory, tactile, bone–conduction auditory, or mixed) and applications (VR or robotic), which have proven the proposed approach validity for the so expected by our society human augmentation solutions.

## 5. Conclusions

The nine reviewed in this paper robotic (humanoid, vehicular, and prosthetic) and VR (a computer graphic walking agent) BCI control studies demonstrated results obtained with the novel tactile and auditory BCI paradigms. The two chosen non-visual spatial sensory modalities did not require any muscle movements (e.g., eye focusing or saccades necessary to operate the majority of visual BCIs). Additionally the VR and robotic devices operation with the spatial tactile or auditory BCI paradigms allowed for the user to focus their sights on the control tasks without the same modality–based stimulus obstruction (the usual case caused by the visual BCIs).

The tactile and especially auditory BCIs usually suffer from lower accuracies. The employed information geometry MDM classifier-based approach served as the very good solution, as shown with the reviewed nine case studies in application to robotic and VR agent control scenarios. The application of the MDM in lower ERP averaging scenarios (1–5) allowed for significant BCI classification accuracy boosts, which approached the perfect average scores for the majority of the improved paradigms. Only the discussed VRtppBCI paradigm (see Section 3.1) resulted on with lower than 99.0% grand mean outcomes (84.1 ± 3.3%) using the five ERP averaging scenarios, yet still above a theoretical threshold of 70% understood as necessary, by the BCI research community, for a smooth communication for locked-in syndrome users (Wolpaw and Wolpaw, 2012).

The results presented in this paper offer a step forward in the maturation of the very promising, as well as very much expected to improve life of ALS patients, human augmentation neurotechnology applications. The evaluated virtual reality and robotic BCI non–visual paradigms, relying on spatial tactile and auditory sensory modalities, still obviously require further improvements or modifications. These needs to determine the major lines of the neurorobotics- and virtual reality-based human augmentation, especially the BCI, research studies for the near future. However, even at the current stage, the proposed neurorobotic and virtual reality BCI applications can be regarded as practical solutions already for the ALS/LIS patients (those locked into their own bodies despite having often intact cognitive functions).

## Author contributions

The author confirms being the sole contributor of this work and approved it for publication.

### Conflict of interest statement

The author declares that the research was conducted in the absence of any commercial or financial relationships that could be construed as a potential conflict of interest.

## Footnotes

^1^An online link to the supplementary material with the vrtbcaBCI and vrtppBCI video https://youtu.be/mLT-CpV5l20

^2^An online link to the supplementary material with the RtcBCI NAO video https://youtu.be/-Qka_AQerLI

^3^An online link to the supplementary material with the btBCI video https://youtu.be/hMEMDCzIk30

^4^An online link to the supplementary material with the RautdBCI video http://youtu.be/JE29CMluBh0

^5^An online link to the supplementary material with the RtppBCI video https://youtu.be/dSrZ5O59vhI

^6^An online link to the supplementary material with the RsaBCI and RtgBCI video https://youtu.be/lKQ8sc_PifE

^7^An online link to the supplementary material with the RfbtBCI NAO video http://youtu.be/sn6OEBBKsPQ
